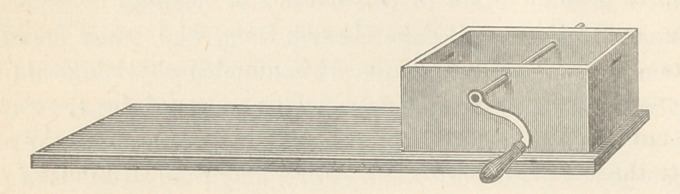# A Practical Bandage Roller

**Published:** 1881-10

**Authors:** Nelson H. Church

**Affiliations:** Chicago


### ARTICLE VIII.

A PRACTICAL BANDAGE ROLLER. By NELSON H. CHURCH, M.D., Chicago.

In the *Medical Record* of July 16 inst., appeared an item entitled "An Easy Way to Roll Bandages." I write to approve of the method, having used the same for ten years. Last winter I thought of improving upon the plan. I made an apparatus, which is not much of an improvement, though it is decidedly more convenient. It is described as follows, viz.: A wooden box, measuring on the inside $2\frac{1}{4}$ x 3 inches, and two inches in depth (or deeper if desired). Two holes were made through the bottom of the box, one in the middle and one near the end. These holes were large enough to admit a wire, as large as a No. 5 catheter. Now take a piece of board one inch thick, four or six inches wide, twelve or eighteen inches long; make two holes in the end and corner of the board to correspond with the holes in the bottom of the box; through these holes put the wire which has previously been bent at right angles—one angle to be half an inch long, the other to be one and a half inch long and to have a thread cut on it (which can be done by a gunsmith); have the short angle go part way through the board, the other to go through and fasten with a screw nut, or, what would perhaps be less trouble, make a groove in the bottom of the board deep enough to accommodate the long end of the wire when bent to the proper angle. This manner of fastening is preferable to screws, because it will not work loose nor split the box. It is not necessary that the box should be fastened to a board; it is only suggested to save mutilating a desk or table. One can sit on this piece of board while rolling the bandages. When one has a table that can be used for the purpose, holes can be bored in one end in the corner, the box fastened and left *in situ* for immediate use. I prefer to have the roller fastened to a table because it is firmer than the board, and one can handle the bandage more readily. The size of the box will admit of rolling bandages wide enough and long enough for any ordinary purposes. The crank is better made of wire the size of a No. 5 catheter and put through the middle of the box, in either diameter, about half an inch from the top; this will admit of rolling a long bandage without touching the bottom.

When rolling a bandage make pressure on the crank, keeping it well in place, and hold the bandage quite firmly over the edge of the box. The end of the bandage should be slightly wetted before commencing to roll, as that makes it adhere to the wire just enough to prevent slipping. After the bandage is wound, pull out the crank, or if the raveling should hold the bandage fast to the wire reverse the crank a few times and withdraw it.

With the smallest amount of practice one can roll a bandage very quickly, very evenly, and very firmly.

---

PRIZE ESSAY.—The Committee of Selection appointed by the chairman of the Section on Practical Medicine, Materia Medica and Physiology, at the recent meeting of the American Medical Association, have selected, and hereby announce, as the subject for the prize to be awarded in 1883, the following question:

What are the special modes of action, or therapeutic effects upon the human system, of water, quinia, and salicylic acid, when used as anti-pyretics in the treatment of disease? The essays must be founded on original experimental and clinical observations, and must be presented to the chairman of the committee of *award* on or before the first day of January, 1883.

N. S. DAVIS,<br>

H. D. HOLTON, } Com. of Selection.<br>

W. B. ULRICH,

## Figures and Tables

**Figure f1:**